# Kawasaki Disease With Acute Acalculous Cholecystitis: A Case Report

**DOI:** 10.7759/cureus.49789

**Published:** 2023-12-01

**Authors:** Nevein F Sejeeni, Sumaiah Alfhmi, Salma Aljahdali, Shroq Alzahrani, Rahaf Jaha

**Affiliations:** 1 General Pediatric, Maternity and Children Hospital, Makkah, SAU; 2 Pediatric Medicine, Maternity and Children Hospital, Makkah, SAU; 3 Internal Medicine, King Abdulaziz Hospital, Makkah, SAU; 4 Medicine and Surgery, Umm Al-Qura University, Makkah, SAU

**Keywords:** systemic vasculitis, five, intravenous immunoglobulin (ivig), coronary artery aneurysms, acute acalculous cholecystitis (aac), kawasaki disease (kd)

## Abstract

Acute acalculous cholecystitis (AAC) is an inflammatory disease of the gallbladder in the absence of gallstones. AAC has been linked to various systemic illnesses including Kawasaki disease (KD). We report a case of a five-year-old male brought to the emergency department (ED) with a history of fever and vomiting for four days. He was admitted as a case of KD. Then, we discovered that he had AAC, which was well managed by intravenous immunoglobulin (IVIG) as the fever subsided, C-reactive protein (CRP) decreased, and repeated abdominal ultrasound showed a decrease in gallbladder thickness without any evidence of coronary artery aneurysms.

## Introduction

Kawasaki disease (KD) is a systemic vasculitis that mostly affects children <5 years of age. Commonly, it manifests with several nonspecific symptoms including abdominal pain, vomiting, diarrhea, rash, cough, runny nose, and irritability [1]. Atypical signs and symptoms would delay clinical recognition, which would lead to a delay in the diagnosis of KD [2]. Gastrointestinal (GI) symptoms are not part of the diagnostic criteria for KD. However, KD patients may initially present with GI or acute surgical abdominal signs and symptoms caused by conditions such as hydrops gallbladder, small intestinal occlusion, abdominal vasculitis, and acute acalculous cholecystitis (AAC) that have been reported in the literature as surgical abdominal complications of KD [3,4]. Acute acalculous cholecystitis (AAC) is a condition characterized by inflammation of the gallbladder without the presence of gallstones. In children, AAC is believed to account for approximately 50%-70% of all cases of acute cholecystitis. Despite this pathology being initially associated with critically ill or post-surgical individuals, the majority of pediatric cases have been observed in the context of various infectious diseases [5]. AAC has been linked to various systemic illnesses including KD [6]. Despite AAC typically following a benign and self-resolving course, there are rare instances where surgical intervention becomes necessary to mitigate the risk of severe complications such as sepsis and mortality [6,7]. AAC is linked to a higher likelihood of developing coronary artery lesions and displaying resistance to intravenous immunoglobulin (IVIG) therapy, which is a fundamental component of the standard treatment protocol for Kawasaki disease (KD) [2,8]. Also, AAC is considered a sign of a severe disease. Therefore, rapid recognition of AAC is crucial as it can indicate the presence of an underlying systemic illness, particularly in children. This is essential to prevent any delays in diagnosing and treating the condition and to effectively mitigate the risk of developing coronary lesions [2].

As far as we know, there is scarce literature discussing cases of KD and AAC. In this article, we report a case involving a pediatric patient who was diagnosed with Kawasaki disease (KD) complicated by AAC. The patient's condition was effectively managed by intravenous immunoglobulin (IVIG) treatment, and no indications of coronary artery aneurysms were observed.

## Case presentation

**Figure 1:**
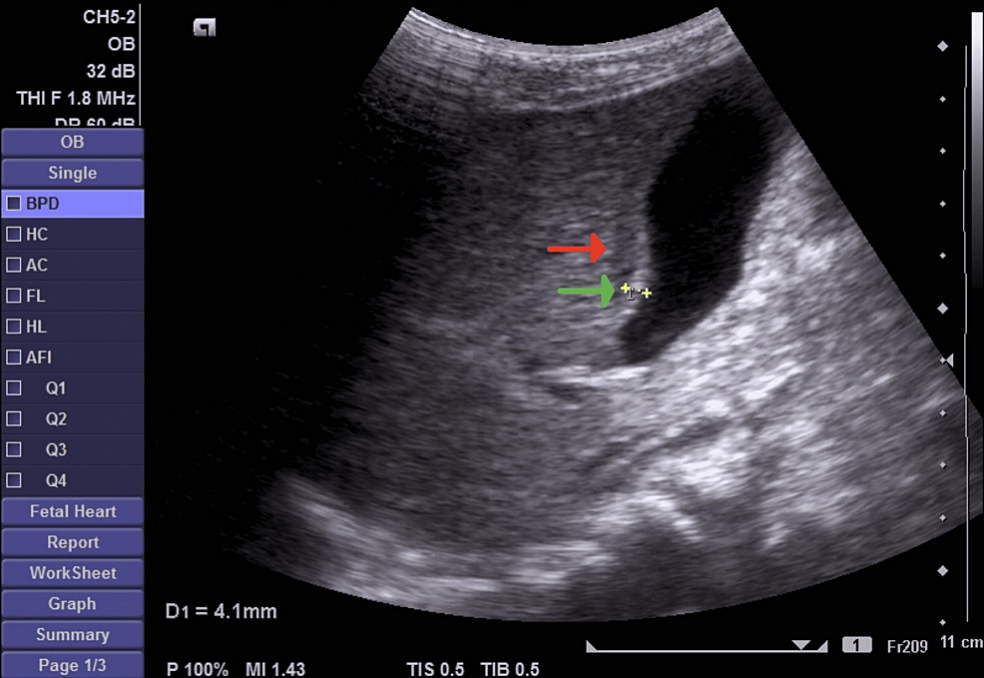
Abdominal ultrasound Red arrow: pericholecystic free fluid, green arrow: gallbladder wall thickness (4 mm)

A five-year-and-six-month-old male, a known case of bronchial asthma on Ventolin and Pulmicort (PRN), was brought to the emergency department (ED) with a fever and vomiting for four days. The fever was documented at 39°C. On arrival at the ED, his vital signs were as follows: temperature of 37.9°C, pulse rate of 140 beats per minute, blood pressure of 84/41 mmHg, and oxygen saturation of 100% on room air. On examination, there was eye puffiness and redness, lip swelling, tongue redness, palm and sole rashes, and tenderness of the right upper abdominal quadrants. Laboratory studies showed white blood cell of 8.7 × 10^3^/UI, hemoglobin of 11.3 g/dL, platelet of 161 × 10^3^/UI, erythrocyte sedimentation rate (ESR) of 78 mm/hour, negative C-reactive protein (CRP), total bilirubin of 9.1 umol/L, direct bilirubin of 5.7 umol/L, aspartate aminotransferase (AST) of 33 IU, alanine transaminase (ALT) of 27 IU, and alkaline phosphatase (ALP) of 137 IU. On imaging, an echocardiogram revealed a minimal pericardial effusion and a normal coronary artery. Also, the abdominal ultrasound revealed minimal free fluid. This patient was admitted as a case of Kawasaki disease. He started on oral high-dose aspirin, IVIG, methylprednisolone as pulse therapy, and maintenance prednisolone. The next day after receiving the treatment, the patient developed abdominal distension, so an abdominal ultrasound was done and showed pericholecystic free fluid, with a gallbladder wall thickness of 4 mm indicating AAC (Figure 1).

A diagnosis of KD with AAC was made, which is a rare presentation. The echocardiogram results revealed no signs of coronary artery aneurysms in his case. Fortunately, all symptoms improved with the treatment. As a result, the patient was discharged from the hospital after a 13-day stay, without experiencing any additional complications.

## Discussion

KD is a condition in which a patient experiences a fever lasting for five or more days (or until the date when intravenous immunoglobulin is administered if given before the fifth day of fever), along with the presence of at least four out of the following five clinical signs: erythema and cracking of lips, strawberry tongue, or erythema of the oral and pharyngeal mucosa; bilateral non-exudative conjunctivitis with peri-limbal sparing; maculopapular rash, diffuse erythroderma, or erythema multiforme-like; erythema and edema of the hands and feet in the acute phase; and cervical lymphadenopathy (≥1.5 cm in diameter), usually unilateral. Patients who exhibit fever and coronary artery abnormalities (CAAs) but do not meet the aforementioned case definition for Kawasaki disease (KD) are categorized as having atypical or incomplete KD [9].

In the United States, Kawasaki disease (KD) is acknowledged as a known contributor to acquired heart disease, with an incidence of 19 cases per 100,000 children under the age of five. The disease can lead to serious complications, including coronary artery dilatations and aneurysms. Echocardiography is the preferred imaging method for identifying coronary artery abnormalities and evaluating myocardial function. Nevertheless, the administration of IVIG and aspirin has significantly decreased the occurrence of coronary lesions in affected children [10,11]. Certain individuals diagnosed with KD may experience gastrointestinal symptoms, including abdominal pain, nausea, vomiting, or hepatobiliary abnormalities reflected in laboratory and radiological tests. These symptoms can initially hide or overlap with typical KD symptoms, leading to misdiagnosis as a gastrointestinal or hepatobiliary disorder such as AAC or hepatitis. Although the etiology and pathogenesis of acute acalculous cholecystitis (AAC) remain unclear, it is documented to have an association with coexisting systemic infections and metabolic abnormalities, as well as other systemic pathologies such as Kawasaki disease (KD). While hepatobiliary complications may not significantly contribute to patient mortality, AAC arising from KD can present diagnostic challenges due to nonspecific clinical symptoms. This may delay the diagnosis of KD, thereby increasing the risk of developing coronary artery complications [7]. To identify AAC, a minimum of two of the subsequent ultrasonic indicators need to be present: enlargement of the gallbladder, a wall thickness exceeding 3.5 mm, the presence of sludge, or the presence of pericholecystic fluid [8]. Regarding laboratory findings, several studies indicate that the Harada score, which includes clinical and laboratory findings, suggests that a high level of total bilirubin can serve as additional evidence for diagnosing coronary artery abnormalities (CAAs), which are strongly associated with AAC [7]. Early detection of AAC is crucial, especially considering the high occurrence of coronary artery aneurysms associated with it, despite its rarity among pediatric patients [7].

## Conclusions

Given that Kawasaki disease patients may present with atypical presentations, it is necessary to consider atypical presentations such as the occurrence of AAC as evident in our patient, which is an atypical case. KD can present with signs and symptoms that diverge from the typical pattern. Therefore, it is crucial to keep KD in mind when dealing with pediatric patients who experience prolonged fever, elevated inflammatory markers, and gastrointestinal issues. A considerable number of patients with KD may develop coronary artery abnormalities, particularly when the diagnosis is overlooked or treatment is delayed. Echocardiography is the imaging modality for identifying coronary artery abnormalities and evaluating myocardial function. Additionally, it plays a valuable role in characterizing and stratifying the risk of patients with KD.
